# Virtual Mutagenesis of the Yeast Cyclins Genetic Network Reveals Complex Dynamics of Transcriptional Control Networks

**DOI:** 10.1371/journal.pone.0018827

**Published:** 2011-04-25

**Authors:** Eliska Vohradska, Jiri Vohradsky

**Affiliations:** 1 Laboratory of Bioinformatics, Institute of Microbiology ASCR v.v.i., Prague, Czech Republic; 2 Institute of Chemical Technology, Faculty of Food and Biochemical Technology, Prague, Czech Republic; Michigan State University, United States of America

## Abstract

Study of genetic networks has moved from qualitative description of interactions between regulators and regulated genes to the analysis of the interaction dynamics. This paper focuses on the analysis of dynamics of one particular network – the yeast cyclins network. Using a dedicated mathematical model of gene expression and a procedure for computation of the parameters of the model from experimental data, a complete numerical model of the dynamics of the cyclins genetic network was attained. The model allowed for performing virtual experiments on the network and observing their influence on the expression dynamics of the genes downstream in the regulatory cascade. Results show that when the network structure is more complicated, and the regulatory interactions are indirect, results of gene deletion are highly unpredictable. As a consequence of quantitative behavior of the genes and their connections within the network, causal relationship between a regulator and target gene may not be discovered by gene deletion. Without including the dynamics of the system into the network, its functional properties cannot be studied and interpreted correctly.

## Introduction

“The identification of network motifs has been widely considered as a significant step toward uncovering the design principles of biomolecular regulatory networks. To date, time-invariant networks have been considered. However, such approaches cannot be used to reveal time-specific biological traits due to the dynamic nature of biological systems and, hence, may not be applicable to development, where temporal regulation of gene expression is an indispensable characteristic”. This sentence is adopted from the paper of Kim et al. [1] and characterizes recent focus in the field of genetic networks – network dynamics and its consequence for their biological function [2]. This topic is also a subject of this paper.

**Figure 1:**
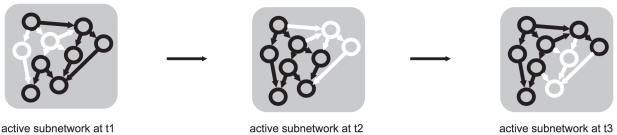
Time varying network motifs.

**Figure 2:**
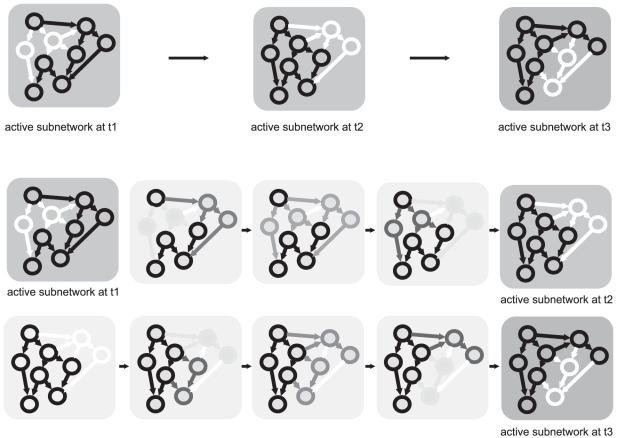
Transition of network structures given by **Figure 1**. Darkness of the gene node and its connection reflects the influence of the regulator to the regulated gene's temporal expression level.

Kim et al. in his paper developed a concept of temporally varying networks. Each time-specific network has its own network motifs and the network motifs change over time (Figure 1). Temporal change of the network structure means that a static network, i.e., the network derived from binding experiments, representing logical relationships between genes (the nodes of the network), is utilized differently at different times during some time-evolving process. If we imagine the dynamic nature of gene expression, where expression of particular genes changes over time, then the different temporal patterns of the networks shown in Figure 1 represent temporal gene expression levels in the form of a network diagram. In principle, Figure 1 can be redrawn to a movie with the snapshots shown in Figure 2. In Figure 2, the shading of a gene node and its connection reflects the influence of the regulator on the temporal expression level of the regulated gene. The concept of varying networks is thus a projection of gene expression dynamics in the form of a directed graph of gene interactions. By examining the temporary gene expression profiles, it is obvious that at a particular moment, the expression of a particular gene can be so low that the connection to this node (gene) is practically functionless. Evolution from one state of the potential network to another over time is graphically depicted in Figure 2. It is obvious from these analyses that the networks derived from static DNA binding experiments are only potential and that their temporal realization depends on the state of gene expression at a given time point [1], [3].

Genetic networks can, in principle, be described by a directed graph. Such modeling invokes a Boolean relationships among the nodes of a network; that is, if gene A is connected with gene B by a logical relationship, then if A is ON, B is also ON (if the relationship is positive) or OFF (if the relationship is negative). For these networks, it is quite easy to calculate terminal states as attractors or basins of attraction, and from this point of view, they have been extensively studied [4], [5], [6], [7], [8], [9]. In the real world, the situation is more complicated because gene expression is, in principle, a set of binding equilibria and biochemical reactions; thus, the expression level of a regulated gene depends on the expression level of the regulator. This notion led to the introduction of logical and threshold functions to the Boolean networks [10], [11], which made Boolean networks more realistic, but it was more difficult to determine the parameter values of a given function. In addition to the Boolean approaches, transcriptional networks have been modeled using a variety of other methods, such as Bayesian networks [12], Petri nets [13] or, recently, Gaussian processes [14], [15]. Genetic network models are summarized in several reviews [16], [17], [18], [19].

Genetic networks represent causal relationships among regulators (transcription factors) and regulated genes, which can also be regulators. Such interaction then form complex networks with feedback and feed forward loops whose topology have been quite extensively studied in recent years [20], [21], [22], [23]. To what extent the dynamics of gene expression can influence the network properties is the subject of this paper.

## Results

### Genetic network model

If we want to formalize transcription control processes so that they can be treated mathematically, then we can start with fundamental molecular interactions that lead to gene transcription. In principal, the probability of occurrence of a gene transcription event is given by the probability of binding of a given transcription factor molecule to the promoter region of a gene. Other molecules can be considered as readily available in sufficient amount, and therefore, referring to the principles of chemical reaction kinetics, the determining factor in the process of transcription is the number of molecules of a particular transcription factor that is present. The probability of binding of the transcription regulator (or regulators) to a given promoter (promoters) is determined by its affinity for the promoter, which is analogous to a binding constant and is often referred to as a promoter strength, and the number of molecules of the regulator. With a low number of regulators molecules, i.e., a low local concentration, the probability of transcription event occurrence is very low and, under a certain threshold, does not occur at all. Transcription starts when the local concentration of the regulator is sufficient, and the rate of transcription grows proportionally to the regulator concentration until a certain level. At this level, the promoter is saturated, and the transcription rate is at its maximum; a further increase in the amount of the regulator does not increase the rate of transcription. The relationship between regulator (regulators) and gene transcript concentrations has therefore a sigmoidal character (sigmoid in biological reactions was thoroughly studied in the work of Veitia [24]). Level of influence, i.e., the affinity for binding of the regulator to DNA, can be expressed as a weight, specific for a given promoter and a regulator (regulators). This simple analysis leads to a formulation of a model where the rate of expression of a given gene transcript is proportional to the regulator concentration and its weight, transferred by a sigmoidal function, and is reduced by degradation. Under this assumption, using an analogy with recurrent neural networks, a simple model of gene expression was derived [25] and extended further in the works of Vu and To et al. [3], [26], [27], [28].
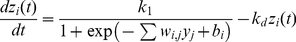
(1)for *n* regulated genes *z_i_*, *i = 1‥n* and *m* transcription factors *y_j_*, *j = 1‥m*, where *k_d_z(t)* is a first order degradation function. *z* represents gene expression levels of regulated gene measured over time *t*. *y* represents expression levels of regulators measured in the same time interval. The influence of each transcription factor is weighted by a constant *w_i,j_*, and *b_i_* represents the reaction delay parameter. The question remains of how to determine the constants *k_1_*, *k_d_*, *w_i,j_*, and *b_i_* from the experimental data. As shown above, the gene expression process and the incidental gene network topology are temporal processes; therefore, the parameters of Eq. 1 can be derived from temporal gene expression measurements. These time series can be measured using high throughput quantitative technologies such as DNA microarrays or qRT-PCR. The parameters can be computed by fitting the measured gene expression profile of the regulated gene **z**_i_ *(t)* to the computed expression profiles 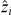 *(t,y,* ***w*** *,b,* ***k*** *)*, minimizing an objective function
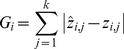
(2)


For target gene *i = 1‥n*, where *z* represents the expression profile measured in *j = 1‥k* time points, and 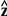 is an expression profile computed by solving equation 1. Without additional information, it would be necessary to compute parameters for all possible combinatorial interactions among all regulators and all regulated genes. This computation is very impractical and, moreover, can lead to a number of false positive results. Fortunately, a number of static measurements exist; defining the potential network by determining which of the regulators can bind to the given promoter and, thus, regulate the given gene. Most of such networks were derived from ChIP-on-chip measurements. Therefore, all interactions not given by these measurements can be excluded from the parameter fitting step. Computing the parameters of individual interactions allows us to formulate “dynamic” models of gene expression networks that not only define interactions among the genes of the network but allow the computation of gene expression levels from the expression levels of other genes in the network – it is possible to study dynamic properties of a network by simulating different experimental conditions. It is possible to make virtual experiments.

### Description of the system

The yeast cell cycle is controlled by many genes, and a fundamental microarray experimental study of this topic on a genome-wide level has been performed by Spellman et al. [29]. It has been documented [30], [31] that the transition between stages of the cell cycle is associated with oscillations in the activity of cell division control protein 28 (CDC28)-cyclin complexes: cyclin synthesis is necessary for phase entry, and CDK-cyclin inhibition/degradation is necessary for phase exit. The G1 and S cyclins CLN1, CLN2, CLB5, and B-type cyclin involved in DNA replication CLB6 accumulate and associate with CDC28 in late G1, the B-type cyclins involved in cell cycle progression CLB1–CLB4 accumulate and associate with CDC28 in G2 and M. These CDK-cyclin complexes can be inhibited by specific cyclin-CDK inhibitors such as SIC1 and FAR1, or can be targeted for degradation by, for example, the anaphase promoting complex [32].

Simon et al. [32], using genome-wide location data and previously reported findings, identified a transcriptional regulatory network for cyclins. The reasons for the choice of the cyclins network were that the network was identified using genome-wide location analysis; the network was relatively small, comprising only 22 genes, and closed; i.e. most of the interactions occurred within the network. The influence of unknown factors from outside the network is thus minimized. There was also a previous experiment with microarrays available that measured expression by sampling relatively densely throughout the yeast cell cycle; this experiment was performed in triplicate allowing for a basic determination of the confidence limits of the measurement [33].

In this paper, we used the yeast cyclins genetic network as a representative case of a gene regulatory network. Together with the microarray kinetic data and ChIP-on-chip measurements, we were able to create a numerical model of this network and analyze its dynamic properties using virtual gene deletion.

### Network reconstruction

**Figure 3:**
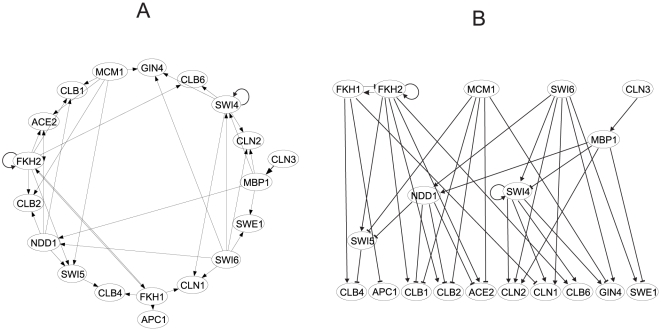
Yeast cyclins network topology reconstructed from binding experiments, gene interactions modeling (**Eq. 1**) and confirmed by literature search. A. Cyclic diagram. B. causal relationship diagram.

The cyclins network analyzed here was reconstructed from the experimental data as described in the Methods. Constraints for the creation of the networks used in the analysis were as follows: 1. interaction between regulators and promoter of the regulator gene had to be confirmed experimentally by ChIP-on-chip experiments, here, we used data published by Simon et al. [32]; 2. the gene expression profile reconstructed using the model had to fall within the 5% confidence interval of the experimentally measured gene expression profiles (experimental gene expression profiles together with the confidence intervals are shown in the Figure S1); and 3. although the inherent experimental and biological variation does not allow for the creation of a single “best” network [3], for the purpose of this paper, we had to chose a single network. Therefore, when constructing the network, we considered only those connections that were previously documented in literature. The resulting network is shown in Figure 3. Panel A shows the wiring diagram; panel B shows the same diagram redrawn to demonstrate the causal connection between the genes of the network. As panel B shows, there exists a first level of genes (FKH1, FKH2, MCM1, SWI6 and CLN3) that are not controlled within the network, and the bottom-most layer (CLB4, APC1, CLB1, CLB2, ACE2, CLN2, CLN1, CLB6, GIN4 and SWE1) represents the terminal nodes of the network, which do not control any other genes in the network. The reconstruction using the model (Eq. 1) allowed us to compute the parameters that best fit the experimental time series for each of the connections. Therefore, the network was fully characterized by the parameters, and by knowing the expression profiles of the first layer, it is possible to directly compute expression profiles of all of the remaining genes.

### Virtual deletion of genes of the first layer (FKH1, FKH2, MCM1, SWI6 and CLN3)

**Figure 4:**
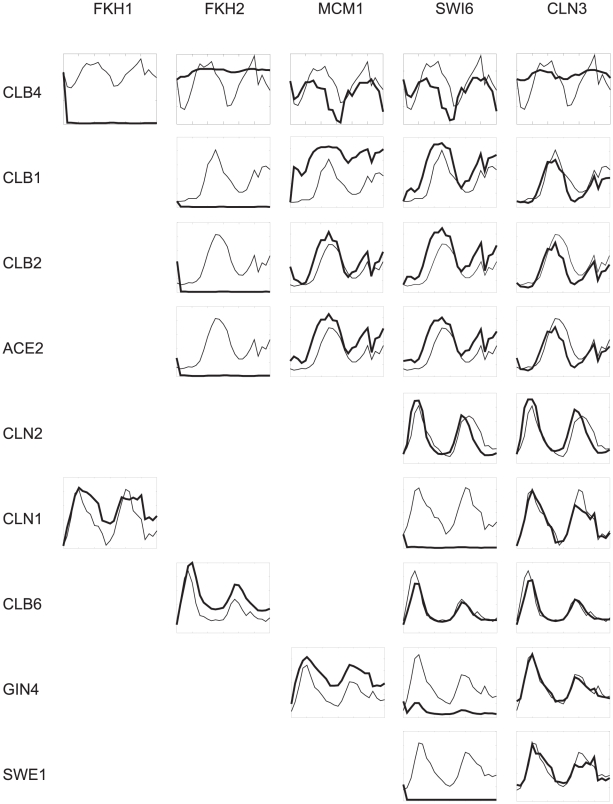
Expression profiles of the genes of the last layer of **Figure 3B** (rows) resulting from the virtual deletion of the genes of the first layer of **Figure 3B** - FKH1, FKH2, MCM1, SWI6 and CLN3 (columns). Thin lines represent measured expression profiles, thick lines represent computed profiles after virtual deletion of genes in the columns.

Virtual gene deletion, which was used to analyze the network dynamics, can be performed by substituting the particular expression profile of a gene in the first layer with a vector of zeros. Impact of the virtual mutation on other genes was determined by computation of their expression profiles using the parameters computed previously. The process of gene deletion was performed one by one for genes of the first layer of Figure 3B. Results are shown in Figure 4 in a matrix of graphs where rows *i* represent genes of the last layer and columns *j* the genes of the first layer. Result of the mutation of the gene *j* for a target gene *i* is in the cell *ij* of the matrix (APC1 was excluded because its control is trivial; it is controlled by only one regulator, FKH1). From Figure 4, it is evident that some gene deletions have a direct impact on the genes of the lowest level of the causal network, for example, FKH2 deletion on the levels of CLB1, CLB2, ACE2 and CLB6 with which FKH2 is directly connected. Importantly, there are connections that are indirect, involving intermediate levels of the genes MBP1, NDD1, SWI4, and SWI5. The control including indirect connections is more complicated. For instance, control of CLB4 is quite complex, and it is, therefore, advisable to consider its control more closely.

**Figure 5:**
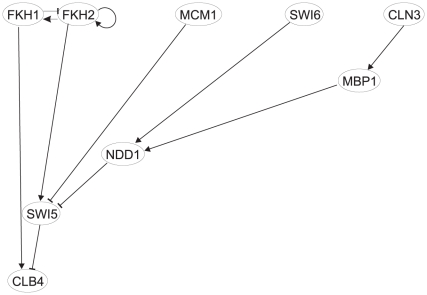
Regulatory pathway of CLB4.

CLB4 is controlled by FKH1 directly and indirectly by MCM1 and FKH2 via SWI5; by SWI6 via NDD1 and SWI5; and by CLN3 via MBP1, NDD1, and SWI5 (see Figure 5). Deletion of FKH1 is quite straightforward because it removes its positive control and results in the repression of the CLB4 expression profile (see Figure 4). Deletion of FKH2 removes periodicity but preserves the mean expression level; the positive control of SWI5 by FKH2 is removed resulting in total repression of SWI5 - the repression force of SWI5, which peaks at approximately 50 h, on CLB4 is removed. The result is the loss of periodicity of CLB4. MCM1 mutation activates SWI5, which then represses CLB4. SWI6 mutation flattens the NDD1 profile. Flat NDD1 profile consequently increases the activity of SWI5, which partially represses CLB4. CLN3 mutation causes MBP1 to be expressed at its basal level, removing the periodicity of NDD1, which results in the loss of periodicity of CLB4. This example is only one illustration of the influence of weighted regulator concentration on the expression level of a target gene. In this case, none of the virtual mutations led to the complete repression of the target gene. If we consider Boolean relationships, then deletion of FKH1 would cause repression of CLB4. For deletion of FKH2, SWI5 would be repressed and CLB4 over-expressed. For MCM1, SWI6, and CLN3, we cannot make a prediction because all of them result in a change of SWI5 control, which cannot be estimated from Boolean rules unless we know a Boolean function for multiple regulators.

Even more striking is the effect of CLN3, which indirectly controls all genes of the bottom layer. However, its deletion has an effect only on CLB4; in all other cases, its deletion is compensated within the pathway (see Figure 4).

If we look more closely on the values of parameter *w* for the connection between MBP1 and other lower-in-cascade genes, it can be seen that it is very low in comparison with the weights of other regulators controlling the given gene ([SWI6; MBP1]→SWE1 [3.5, **−0.14**]; [SWI6; SWI4; MBP1]→SWI4 [0.14, 1.37, **−0.19**]; [MBP1; SWI6]→NDD1 [**0.92**, 3.4]; [SWI6;SWI4;MBP1]→CLN2 [2.80,1.03,**0.01**], for comparison, *w* of connections is noted in the square brackets, *w_MBP1_* is given in bold), its deletion therefore does not influence expression profiles of the genes in the lower most level. Although the ChIP-on-chip data indicate possible binding of CLN3 to the promoter of MBP1, the *w* computed for this connection is also very low. Deletion (as well as overexpression, data not shown) of CLN3 therefore does not influence other genes. Literature search indeed indicated posttranslation control instead of the transcriptional control [34]. MBP1 was reported as regulated by CLN3 (indirectly by means of WHI5). As the presented model does not include posttranscriptional events, such connection cannot be discovered and the low value of w_CLN3-MBP1_ is quite justified. In contrast, the low value of *w_MBP1-other genes_* has low influence upon deletion, but overexpression of MBP1 (data not shown) has pronounced and divergent effect on the genes lower in cascade. Therefore this connection is, in comparison with CLN3-MBP1 interaction, meaningful.

Another similar example is the influence of deletion of SWI6 on the expression of CLN2 and CLB6. Both genes are controlled through SWI4, and SWI4 is the dominant regulator of the genes. For SWI4, the most important control effect is its self-induction; therefore, deletion of SWI6 has almost no effect on its expression, resulting in the loss of the deletion effect of SWI6 on the expression levels of CLN2 and CLB6. For CLN1, which is also controlled by SWI4 and SWI6, the effect of SWI4 is low, and deletion of SWI6 causes the complete repression of CLN1 by strong FKH1. The same effect is observed for GIN4, where the repressor is MCM1 instead of FKH1.

### Virtual deletion of genes of the second layer (SWI5, NDD1, SWI4 and MBP1)

**Figure 6:**
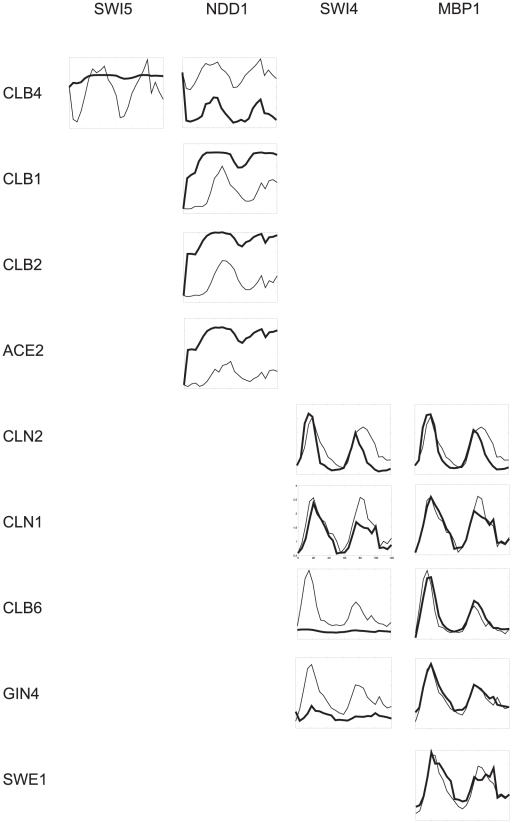
Expression profiles of the genes of the last layer of **Figure 3B** (rows) resulting from the virtual deletion of the genes of the intermediate layer of **Figure 3B** - SWI5, NDD1, SWI4 and MBP1 (columns). Thin lines represent measured expression profiles, thick lines represent computed profiles after virtual deletion of genes in the columns.

Influence of the deletion of the genes of intermediate level of the causal network (Figure 3B, SWI5, NDD1, SWI4 and MBP1) to the kinetic profiles of the genes of the last layer is shown in Figure 6. Deletion of SWI5 influences only CLB4. By deletion of SWI5, its repressive influence on CLB4 is removed, resulting in increase of the expression level of CLB4. Deletion of NDD1, which represses CLB1, CLB2 and ACE2 has a straightforward effect on their expression. NDD1 deletion increases their expression levels. NDD1 indirectly controls CLB4 by means of SWI5 for which it acts as repressor. Deletion of NDD1 increases levels of SWI5 which consequently represses more CLB4 resulting in overall decrease of its expression level. SWI4 acts as an activator for the genes lover in regulatory cascade (CLN2, CLN1, CLB6 and GIN4). Deletion of SWI4 influences expression of CLB6 and GIN4. By SWI4 deletion, its positive regulatory effect is removed and the expression levels of CLB6 and GIN4 decrease. The influence of SWI4 on CLN1 and CLN2 expression is lower and its deletion is therefore compensated by other regulators controlling these two genes. Consistently with the observations made for CLN3, the deletion of MBP1 has minimal effect on the genes downstream the cascade (CLN2, CLN1, CLB6, GIN4 and SWE1) as *w_MBP1-other genes_* is lower in comparison with the *w* of regulators controlling the affected genes (see above).

A conclusion from these observations is that the effect of regulator concentration and weight determine the expression level of its target genes at the bottom of the regulatory cascade in a highly unpredictable manner, which does not follow simple logic. In certain cases, the deletion of a gene on top of the cascade can have a striking effect (as in the case of the effect of deletion of FKH2 on CLB4), whereas in other cases, this effect completely vanishes in the regulatory cascade, as in the case of genes controlled indirectly by CLN3. An important consequence of the quantitative network behavior is that existing causal relationships between regulators and regulated genes in a cascade may not be discovered by gene deletion. Even direct connections, as in the case of SWI6-CLN2, may not be discovered if the influence of the regulator is not sufficiently pronounced. This statement has a profound consequence on the interpretation of mutagenesis experiments. In principle, we can state that the causal relationship between a regulator and target gene exists if we see the effect of gene deletion; however, we cannot say the opposite: that if the effect is not observed, then the connection does not exist. A relationship may exist but may not be observable. Building conclusions about network topology by drawing links between genes whose causal relationship was discovered by mutation experiments can lead to incomplete and sometimes even incorrect connections. Without including the dynamics of the system into the network, its functional properties cannot be studied and interpreted correctly. Logical interpretation of observations can be completely wrong when the regulation is complicated and includes a cascade of reactions. Therefore, to discover regulatory relationships in genetic networks, one cannot rely solely upon static data but must also consider the dynamics of the network.

## Discussion

Using a mathematical description of regulation of gene expression (Equation 1) and a procedure for computation of its parameters from experimental data, it was possible to construct a complete numerical model of the genetic network of yeast cyclins active during cell cycle. The model was able to fully describe the kinetics of gene expression of any gene of the reconstructed network coherently with the measured gene expression profiles. The model allowed the simulation of a situation when genes in the topmost level of the regulatory cascade were deleted, which simulated experimental gene deletion. Influence of such deletion on the change in the expression profiles of other genes of the network was analyzed. The virtual gene deletion showed that in more complicated cascades of regulation, with many genes in between the deleted gene and the target gene, the result of gene deletion is quite unpredictable and, in several cases, the absence of the deleted gene can be compensated within the cascade. This compensation means that even if there is a causal relationship between the deleted gene and the target gene, it may not be discovered by the mutation experiment.

Conclusions drawn from the dynamic model of the cyclins genetic network can be criticized because they are not experimentally verified. Although this model has been verified by comparison with previously measured experimental data, this point cannot be ignored. If the network model is wrong in particular connections, then parameters that were computed to fit experimentally measured expression profiles would be wrong as well; thus, the results of virtual gene deletion could also be wrong or, at least, altered. A crucial point of this paper is that the response to mutation of genes in the topmost layer has highly unpredictable impact on the genes lower in the causal cascade and that the effect of mutation can disappear in the cascade of reactions. This statement remains unchanged even if the model is, in certain cases, wrong. An error would influence interpretation of a particular network of cyclins, what is indeed, with currently available data possible, but would not change the basic conclusion that the network dynamic is essential in the interpretation of its biological function.

Another point of discussion is the linearity of the relationship between mRNA and final protein concentration. Recent studies show [35] that, as measured by microarrays or qPCR, almost 50% of genes exhibit a linear relationship whereas others are either posttranslationally modified to alter their activity or their relationship is nonlinear for other reasons. For this reasons we excluded from our analysis genes that are known to be controlled postranscriptionally (e.g., according to Bahler et al. [34] CLB2 is controlled transcriptionally by MCM1/FKH2/NDD1 and posttranslationally by CLN1 and SIC1; thus, this connection was excluded).

Any model is only an approximation of the actual biological process and has a value if it can capture its features on a given level of abstraction. We are convinced and provide evidence here that the conclusions drawn in the Results are relevant to the level of abstraction used here. As support for the presented model of the yeast cyclins network, we must emphasize that only connections which are physically possible (i.e., were measured by ChIP-on-chips) and were confirmed by the literature were considered in the final network. The resulting network is thus smaller than the one suggested by Simon et al. [32]. In our previous work [26], [27], [28] and in the work of others [36] it was shown that the assumptions on which the model is based are relevant to the conclusions which are drawn from the simulation presented here. Therefore the conclusions about functioning of dynamic genetic networks can be, with a high level of confidence, considered as valid.

It can be concluded that for the correct interpretation of the biological function of genetic networks dynamic properties cannot be ignored, and that a static network represents only a potential which is utilized differently over time and during different physiological processes.

## Materials and Methods

### Inference of the parameters of the gene expression model

Reconstruction of the topology of the cyclins genetic network was performed using gene expression data published by Pramila et al. [33]. The triplicate experiments in the time series were averaged and used to compute parameters of Eq. 1. Relative mRNA expression levels 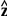 (Eq. 2) can be computed by integrating Eq. 1, provided that the temporal expression profile of the regulator (regulators) and the parameters ***w*** *,b,k_1_,k_d_* are known. If there is not a feedback loop between the regulator and the target gene, then the first term of Eq. 1 remains constant, and Eq. 1 can be integrated (see Text S1). Where this condition was not satisfied, Eq. 1 was solved numerically using a modified Runge-Kutta algorithm as coded in the Matlab ode45 function. Parameters ***w*** *,b,k_1_,k_d_* were computed iteratively with a simulated annealing scheme [37] by minimizing the value of the objective function *G* (Eq. 2) and using gene expression profiles of the cyclins measured by Pramila et al.. Simulated annealing allowed the discovery of a global minimum in a parameter space given by the parameters of Eq. 1 and the objective function *G* (Eq. 2). Parameter optimization was performed 100 times for each connection for different randomly set initial values of the parameters. The parameters giving the smallest value of function *G* were selected for further analysis. Parameters of Eq. 1 were computed for all connections predicted by the ChIP-on-chip experiments. Interactions for which the modeled profiles fell within the 5% confidence interval of the measured gene expression profiles were selected. These interactions were compared with literature, and only those confirmed by previous experiments were selected. The resulting network is shown in Figure 3. Computed parameters allowed for a full reconstruction of the cyclins network at any point within the confidence interval given by the measurement error (for the measured and reconstructed gene expression profiles see Figure S1).

Virtual gene deletion was performed for all genes of the first level (see Figure 3B) individually by substituting the gene expression profile with a vector of zeros. Expression profiles of the remaining genes in the causal cascade of figure 3B were computed using the optimized parameters. Computed profiles of the genes of the lowest level of the cascade are shown in Figure 4 for each individual virtual deletion.

## Supporting Information

Figure S1 Measured expression profiles of the cyclins network.
(EPS)

Text S1 Integration of the model given by Eq. 1.
(DOC)
