# Supplementary material for: Virtual Mutagenesis of the Yeast Cyclins Genetic Network Reveals Complex Dynamics of Transcriptional Control Networks
Source: PLoS One. 2011 Apr 25;6(4):e18827. doi: 10.1371/journal.pone.0018827 (PMC3081828; doi:10.1371/journal.pone.0018827)
Supplement: Text S1 — Integration of the model given by Eq. 1. (DOC) [file pone.0018827.s002.doc]

Integration of the model given by Eq. 1

$$\left\{\begin{matrix} \frac{dz}{dt}=k_{1}\frac{1}{1+\text{Exp}[-(\sum w_{i}y_{i}+b)]}-k_{2}z \\ z(t=0)=z_{0} \end{matrix}\right.$$

Let $A=k_{1}\frac{1}{1+\text{Exp}[-(\sum w_{i}y_{i}+b)]}$,

$$\frac{dz}{dt}=A-k_{2}z\Leftrightarrow\frac{dz}{A-k_{2}z}=dt\Leftrightarrow\int\frac{dz}{A-k_{2}z}=\int dt$$

| $t=-\frac{1}{k_{2}}\ln(A-k_{2}z)+C$ | A1 |
| --- | --- |

$$tk_{2}=-\ln(A-k_{2}z)+C\Leftrightarrow\ln(A-k_{2}z)=-tk_{2}+C\Leftrightarrow A-k_{2}z=e^{(-tk_{2}+C)}$$

| $\Rightarrow z=\frac{A-e^{(-tk_{2}+C)}}{k_{2}}\text{ }$ | A2 |
| --- | --- |

From (A1) and z(t = 0) = z_0_ $\Rightarrow0=-\frac{1}{k_{2}}\ln(A_{0}-k_{2}z_{0})+C\Rightarrow C=\frac{1}{k_{2}}\ln(A_{0}-k_{2}z_{0})$

Equation A2 was used to compute target gene expression profiles.
